# Cavity-based architecture to preserve quantum coherence and entanglement

**DOI:** 10.1038/srep13843

**Published:** 2015-09-09

**Authors:** Zhong-Xiao Man, Yun-Jie Xia, Rosario Lo Franco

**Affiliations:** 1 Shandong Provincial Key Laboratory of Laser Polarization and Information Technology, Department of Physics, Qufu Normal University, Qufu 273165, China; 2 Dipartimento di Fisica e Chimica, Università di Palermo, via Archirafi 36, 90123 Palermo, Italy; 3 Instituto de Física de São Carlos, Universidade de São Paulo, CP 369, 13560-970 São Carlos, SP, Brasil; 4 School of Mathematical Sciences, The University of Nottingham, University Park, Nottingham NG7 2RD, United Kingdom

## Abstract

Quantum technology relies on the utilization of resources, like quantum coherence and entanglement, which allow quantum information and computation processing. This achievement is however jeopardized by the detrimental effects of the environment surrounding any quantum system, so that finding strategies to protect quantum resources is essential. Non-Markovian and structured environments are useful tools to this aim. Here we show how a simple environmental architecture made of two coupled lossy cavities enables a switch between Markovian and non-Markovian regimes for the dynamics of a qubit embedded in one of the cavity. Furthermore, qubit coherence can be indefinitely preserved if the cavity without qubit is perfect. We then focus on entanglement control of two independent qubits locally subject to such an engineered environment and discuss its feasibility in the framework of circuit quantum electrodynamics. With up-to-date experimental parameters, we show that our architecture allows entanglement lifetimes orders of magnitude longer than the spontaneous lifetime without local cavity couplings. This cavity-based architecture is straightforwardly extendable to many qubits for scalability.

Entangled states are not only an existing natural form of compound systems in the quantum world, but also a basic resource for quantum information technology^1^,^2^,^3^. Due to the unavoidable coupling of a quantum system to the surrounding environment, quantum entanglement is subject to decay and can even vanish abruptly, a phenomenon known as early-stage disentanglement or entanglement sudden death^4^,^5^,^6^,^7^,^8^,^9^,^10^,^11^,^12^,^13^. Harnessing entanglement dynamics and preventing entanglement from disappearing until the time a quantum task can be completed is thus a key challenge towards the feasibility of reliable quantum processing^14^,^15^.

So far, a lot of researches have been devoted to entanglement manipulation and protection. A pure maximally entangled state can be obtained from decohered (partially entangled mixed) states^16^,^17^,^18^,^19^,^20^ provided that there exist a large number of identically decohered states, which however will not work if the entanglement amount in these states is small. *In situ*ations where several particles are coupled to a common environment and the governing Hamiltonian is highly symmetric, there may appear a decoherence-free subspace that does not evolve in time^21^,^22^,^23^: however, in this decoherence-free subspace only a certain kind of entangled state can be decoupled from the influence of the environment^24^,^25^. The quantum Zeno effect^26^ can also be employed to manipulate decoherence process but, to prevent considerable degradation of entanglement, special measurements should be performed very frequently at equal time intervals^24^,^25^. By encoding each physical qubit of a many-qubit system onto a logical one comprising several physical qubits^27^,^28^,^29^,^30^,^31^, an appropriate reversal procedure can be applied to correct the error induced by decoherence after a multiqubit measurement that learns what error possibly occurred. Yet, as has been shown^31^, in some cases this method can indeed delay entanglement degradation but in other cases it leads to sudden disentanglement for states that otherwise disentangle only asymptotically. The possibility to preserve entanglement via dynamical decoupling pulse sequences has been also theoretically investigated recently for finite-dimensional or harmonic quantum environments^32^,^33^,^34^,^35^ and for solid state quantum systems suffering random telegraph or 1/*f* noise^36^,^37^, but these procedures can be demanding from a practical point of view.

In general, environments with memory (so-called non-Markovian) suitably structured constitute a useful tool for protecting quantum superpositions and therefore the entanglement of composite systems^8^,^38^,^39^,^40^. It is nowadays well-known that independent qubits locally interacting with their non-Markovian environments can exhibit revivals of entanglement, both spontaneously during the dynamics^38^,^41^,^42^,^43^,^44^ and on-demand by local operations^45^,^46^. These revivals, albeit prolonging the utilization time of entanglement, however eventually decay. In several situations, the energy dissipations of individual subsystems of a composite system are responsible for disentanglement. Therefore, methods that can trap system excited-state population would be effective for entanglement preservation. A stationary entanglement of two independent atoms can be in principle achieved in photonic crystals or photonic-band-gap materials^47^,^48^ if they are structured so as to inhibit spontaneous emission of individual atoms. This spontaneous emission suppression induced by a photonic crystal has been so far verified experimentally for a single quantum dot^49^ and its practical utilization for a multi-qubit assembly appears far from being reached. Quantum interference can also be exploited to quench spontaneous emission in atomic systems^50^,^51^ and hence used to protect two-atom entanglement provided that three levels of the atoms can be used^52^. Since the energy dissipations originate from excited state component of an entangled state, a reduction of the weight of excited-state by prior weak measurement on the system before interacting with the environment followed by a reversal measurement after the time-evolution proves to be an efficient strategy to enhance the entanglement^53^,^54^,^55^. However, the success of this measurement-based strategy is always conditional (probability less than one)^53^,^54^,^55^. It was shown that steady-state entanglement can be generated if two qubits share a common environment^24^,^56^, interact each other^57^ and are far from thermal equilibrium^58^,^59^,^60^,^61^,^62^. It has been also demonstrated that non-Markovianity may support the formation of stationary entanglement in a non-dissipative pure dephasing environment provided that the subsystems are mutually coupled^63^.

Separated, independent two-level quantum systems at thermal equilibrium, locally interacting with their own environments, are however the preferable elements of a quantum hardware in order to accomplish the individual control required for quantum information processing^14^,^15^. Therefore, proposals of strategies to strongly shield quantum resources from decay are essential within such a configuration. Here we address this issue by looking for an environmental architecture as simple as possible which is able to achieve this aim and at the same time realizable by current experimental technologies. In particular, we consider a qubit embedded in a cavity which is in turn coupled to a second cavity and show that this basic structure is able to enable transitions from Markovian to non-Markovian regimes for the dynamics of the qubit just by adjusting the coupling between the two cavities. Remarkably, under suitable initial conditions, this engineered environment is able to efficiently preserve qubit coherence and, when extended to the case of two noninteracting separated qubits, quantum entanglement. We finally discuss the effectiveness of our cavity-based architecture by considering experimental parameters typical of circuit quantum electrodynamics^15^,^64^, where this scheme can find its natural implementation.

## Results

Our analysis is divided into two parts. The first one is dedicated to the single-qubit architecture which shall permit us to investigate the dynamics of quantum coherence and its sensitivity to decay. The second part treats the two-qubit architecture for exploring to which extent the time of existence of quantum entanglement can be prolonged with respect to its natural disappearance time without the proposed engineered environment.

### Single-qubit coherence preservation

**Figure 1:**
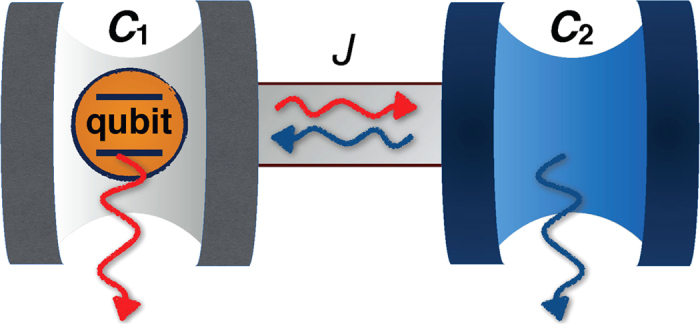
Scheme of the single-qubit architecture. A two-level atom (qubit) is embedded in a cavity *C*_1_ which is in turn coupled to a second cavity *C*_2_ by a coupling strength *J*. Both cavities are taken at zero temperature and can lose photons.

The global system is made of a two-level atom (qubit) inside a lossy cavity *C*_1_ which in turn interacts with another cavity *C*_2_, as depicted in Fig. 1. The Hamiltonian of the qubit and two cavities is given by (*ħ* = 1)


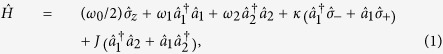


where 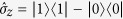 is a Pauli operator for the qubit with transition frequency *ω*_0_, 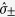 are the raising and lowering operators of the qubit, 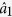 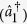 and 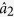 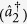 the annihilation (creation) operators of cavities *C*_1_ and *C*_2_ which sustain modes with frequency *ω*_1_ and *ω*_2_, respectively. The parameter *κ* denotes the coupling of the qubit with cavity *C*_1_ and *J* the coupling between the two cavities. We take *ω*_1_ = *ω*_2_ = *ω* and, in order to consider both resonant and non-resonant qubit-*C*_1_ interactions, *ω*_0_ = *ω*+*δ* with *δ* being the qubit-cavity detuning. Taking the dissipations of the two cavities into account, the density operator *ρ*(*t*) of the atom plus the cavities obeys the following master equation^65^


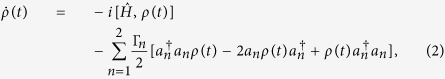


where 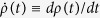 and Γ_1_ (Γ_2_) denotes the photon decay rate of cavity *C*_1_ (*C*_2_). The rate Γ_*n*_/2 physically represents the bandwidth of the Lorentzian frequency spectral density of the cavity *C*_*n*_, which is not a perfect single-mode cavity^65^. A cavity with a high quality factor will have a narrow bandwidth and therefore a small photon decay rate. Weak and strong coupling regimes for the qubit-*C*_1_ interaction can be then individuated by the conditions 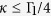 and 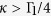
41,65.

Let us suppose the qubit is initially in the excited state 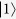 and both cavities in the vacuum states 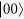, so that the overall initial state is 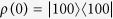, where the first, second and third element correspond to the qubit, cavity *C*_1_ and cavity *C*_2_, respectively. Since there exist at most one excitation in the total system at any time of evolution, we can make the ansatz for *ρ*(*t*) in the form


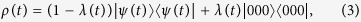


where 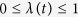 with 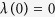 and 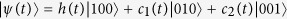 with *h*(0) = 1 and 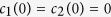. It is convenient to introduce the unnormalized state vector^66^,^67^


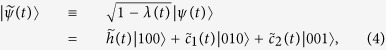


where 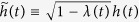 represents the probability amplitude of the qubit and 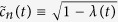 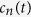 (*n* = 1, 2) that of the cavities being in their excited states. In terms of this unnormalized state vector we then get


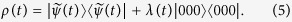


The time-dependent amplitudes 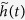, 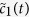, 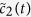 of Eq. (4) are determined by a set of differential equations as


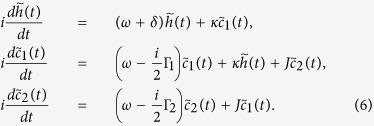


The above differential equations can be solved by means of standard Laplace transformations combined with numerical simulations to obtain the reduced density operators of the atom as well as of each of the cavities. In particular, in the basis 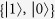 the density matrix evolution of the qubit can be cast as


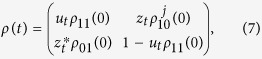


where *u*_*t*_ and *z*_*t*_ are functions of the time *t* (see Methods).

An intuitive quantification of quantum coherence is based to the off-diagonal elements of the desired quantum state, being these related to the basic property of quantum interference. Indeed, it has been recently shown^68^ that the functional


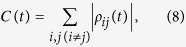


where *ρ*_*ij*_(*t*) (*i* ≠ *j*) are the off-diagonal elements of the system density matrix, satisfies the physical requirements which make it a proper coherence measure^68^. In the following, we adopt C  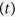 as quantifier of the qubit coherence and explore how to preserve and even trap it under various conditions. To this aim, we first consider the resonant atom-cavity interaction and then discuss the effects of detuning on the dynamics of coherence.

**Figure 2:**
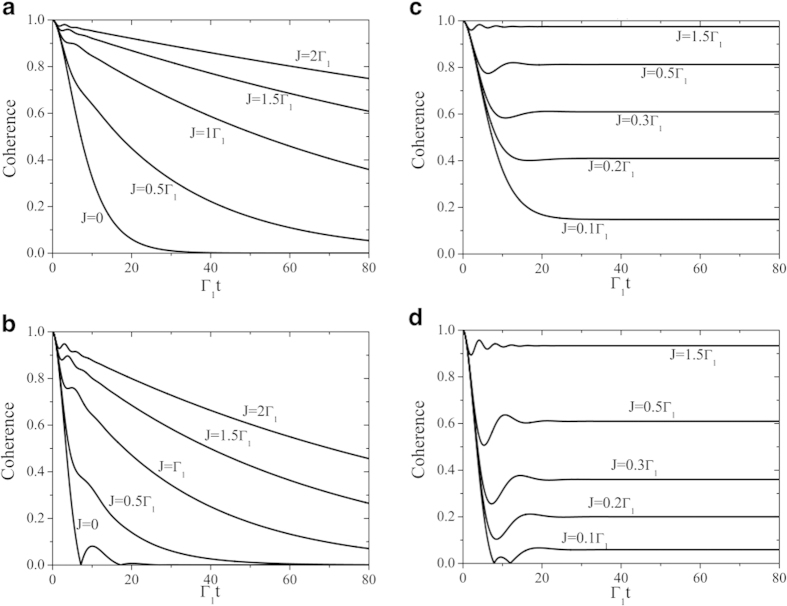
Coherence C 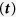 of the qubit as a function of the scaled time Γ_1_*t* for different coupling strengths *J* between the two cavities for (a) *κ* = 0.24 Γ_1_, Γ_2_ = 0.5 Γ_1_ and (b) *κ* = 0.4 Γ_1_, Γ_2_ = 0.5 Γ_1_. The qubit is initially prepared in the state 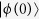 with 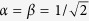 and resonant with the cavity (detuning *δ* = 0). The plots in panels (**c**,**d**) display the coherence trapping for a perfect cavity (Γ_2_ = 0) with *κ* = 0.24 Γ_1_ and *κ* = 0.4 Γ_1_, respectively.

Suppose the qubit is initially prepared in the state 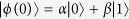 (with 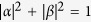), namely, C  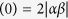, then at time *t* > 0 the coherence becomes C  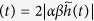. Focusing on the weak coupling between the qubit and the cavity *C*_1_ with *κ* = 0.24 Γ_1_, we plot the dynamics of coherence in Fig. 2(a). In this case, the qubit exhibits a Markovian dynamics with an asymptotical decay of the coherence in the absence of the cavity *C*_2_ (with *J* = 0). However, by introducing the cavity *C*_2_ with a sufficiently large coupling strength, quantum coherence undergoes non-Markovian dynamics with oscillations. Moreover, it is readily observed that the decay of coherence can be greatly inhibited by increasing the *C*_1_-*C*_2_ coupling strength *J*. On the other hand, if the coupling between the atom and the cavity *C*_1_ is initially in the strong regime with the occurrence of coherence collapses and revivals, the increasing of the *C*_1_-*C*_2_ coupling strength *J* can drive the non-Markovian dynamics of the qubit to the Markovian one and then back to the non-Markovian one, as shown in Fig. 2(b). This behavior is individuated by the suppression and the successive reactivation of oscillations during the dynamics. It is worth noting that, although the qubit can experience non-Markovian dynamics again for large enough *J*, the non-Markovian dynamics curve is different from the original one for *J* = 0 in the sense that the oscillations arise before the coherence decays to zero. In general, the coupling of *C*_1_-*C*_2_ can enhance the quantum coherence also in the strong coupling regime between the qubit and the cavity *C*_1_.

**Figure 3:**
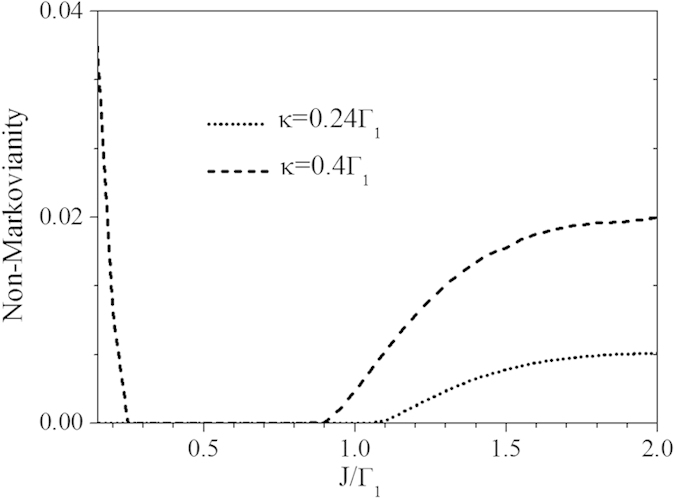
Non-Markovianity quantifier 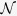 of Eq. (9) of the qubit dynamics as a function of *J*/Γ_1_ for weak (*κ* = 0.24 Γ_1_) and strong (*κ* = 0.4 Γ_1_) coupling regimes to cavity *C*_1_ and a fixed decay rate Γ_2_ = 0.5 Γ_1_ of the cavity *C*_2_.

The oscillations of coherence, in clear contrast to the monotonic smooth decay in the Markovian regime, constitute a sufficient condition to signify the presence of memory effects in the system dynamics, being due to information backflow from the environment to the quantum system^69^. The degree of a non-Markovian process, the so-called non-Markovianity, can be quantified by different suitable measures^69^,^70^,^71^,^72^. We adopt here the non-Markovianity measure which exploits the dynamics of the trace distance between two initially different states *ρ*_1_(0) and *ρ*_2_(0) of an open system to assess their distinguishability^69^. A Markovian evolution can never increase the trace distance, hence nonmonotonicity of the latter would imply a non-Markovian character of the system dynamics. Based on this concept, the non-Markovianity can be quantified by a measure 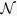 defined as^69^


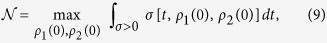


where 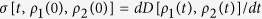 is the rate of change of the trace distance, which is defined as 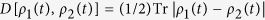, with 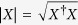. By virtue of 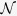, we plot in Fig. 3 the non-Markovianity of the qubit dynamics for the conditions considered in Fig. 2(a,b). We see that if the qubit is initially weakly coupled to the cavity *C*_1_ (*κ* = 0.24 Γ_1_) its dynamics can undergo a transition from Markovian 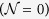 to non-Markovian 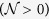 regimes by increasing the coupling strengths *J* between the two cavities. On the other hand, for strong qubit-cavity coupling (*κ* = 0.4 Γ_1_), the non-Markovian dynamics occurring for *J* = 0 turns into Markovian and then back to non-Markovian by increasing *J*. We mention that such a behavior has been also observed in a different structured system where a qubit simultaneously interacts with two coupled lossy cavities^73^.

Trapping qubit coherence in the long-time limit is a useful dynamical feature for itself that shall play a role for the preservation of quantum entanglement to be treated in the next section. We indeed find that the use of coupled cavities can achieve this result if the cavity *C*_2_ is perfect, that is Γ_2_ = 0 (no photon leakage). The plots in Fig. 2(c,d) demonstrate the coherence trapping in the long-time limit for both weak and strong coupling regimes between the qubit and the cavity *C*_1_ for different coupling strengths *J* between the two cavities. This behavior can be explained by noticing that there exists a bound (decoherence-free) state of the qubit and the cavity *C*_2_ of the form 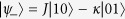, with *J* and *κ* being the *C*_1_-*C*_2_ and qubit-*C*_1_ coupling strengths. Being this state free from decay, once the reduced initial state of the qubit and the cavity *C*_2_ contains a nonzero component of this bound state 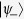, a long-living quantum coherence for the qubit can be obtained. For the initial state 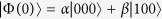 of the qubit and two cavities here considered and Γ_2_ = 0, the coherence defined in Eq. (8) gets the asymptotic value C   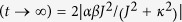, which increases with *J* for a given *κ*. We further point out that the cavity *C*_1_ acts as a catalyst of the entanglement for the hybrid qubit-*C*_2_ system, in perfect analogy to the stationary entanglement exhibited by two qubits embedded in a common cavity^24^. In the latter case, in fact, the cavity mediates the interaction between the two qubits and performs as an entanglement catalyst for them.

**Figure 4:**
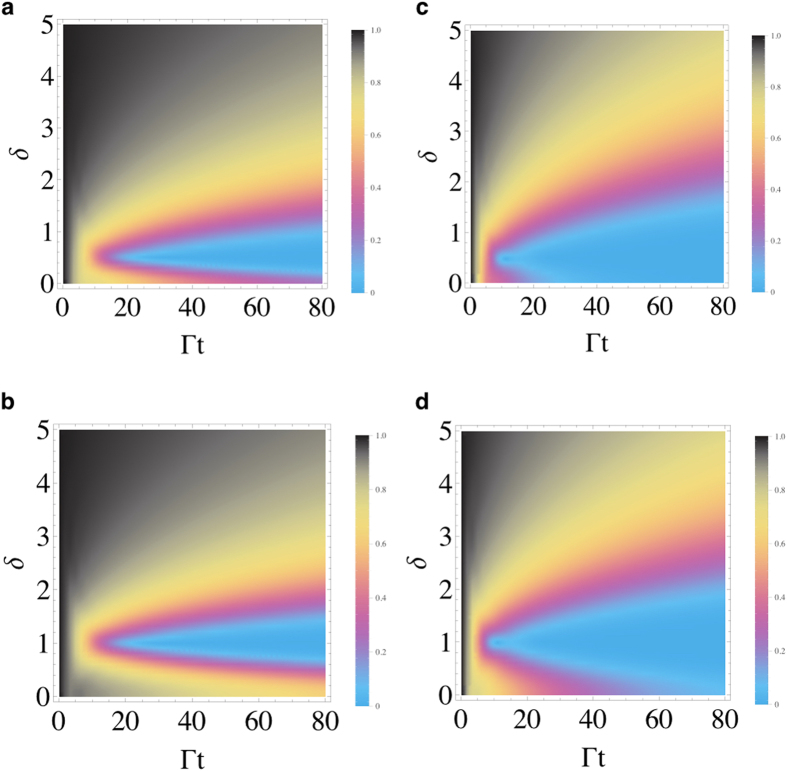
Density plots of coherence C   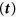 of the qubit as functions of detuning *δ* and the scaled time Γ_1_*t* for (a) *κ* = 0.24 Γ_1_, Γ_2_ = 0.2 Γ_1_, *J* = 0.5 Γ_1_; (b) *κ* = 0.24 Γ_1_, Γ_2_ = 0.2 Γ_1_, *J* = Γ_1_; (c) *κ* = 0.4 Γ_1_, Γ_2_ = 0.5 Γ_1_, *J* = 0.5 Γ_1_; (d) *κ* = 0.4 Γ_1_, Γ_2_ = 0.5 Γ_1_, *J* = Γ_1_. The initial state of the qubit is maximally coherent 
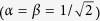
. The values of the coherence are within the range: [0, 1].

**Figure 5:**
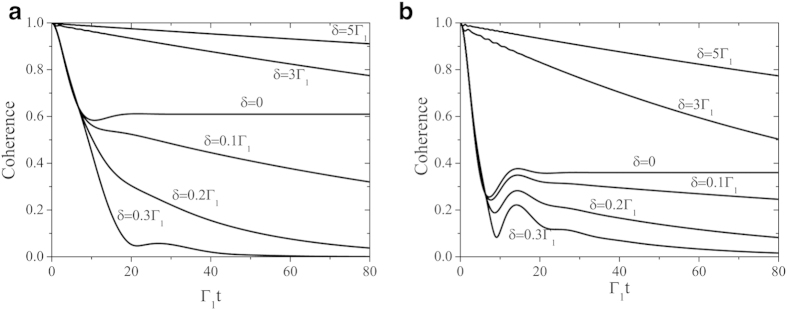
Coherence C   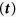 of the qubit as a function of the scaled time Γ_1_*t* for different values of the detuning *δ* in the case when the cavity *C*_2_ is perfect, that is Γ_2_ = 0. The qubit-*C*_1_ and the *C*_1_-*C*_2_ coupling strengths are, respectively, (**a**) *κ* = 0.24 Γ_1_, *J* = 0.3 Γ_1_; (**b**) *κ* = 0.4 Γ_1_, *J* = 0.3 Γ_1_. Out of resonance (*δ* > 0) no coherence trapping is achievable.

We now discuss the effect of non-resonant qubit-*C*_1_ interaction (*δ* ≠ 0) on the dynamics of coherence. In Fig. 4(a–d), we display the density plots of the coherence as functions of detuning *δ* = *ω*_0_ − *ω* and rescaled time Γ*t* for both weak and strong couplings. One observes that when *δ* departures from zero, the decay of coherence speeds up achieving the fastest decay around *δ* = *J*. It is interesting to highlight the role of the cavity-cavity coupling parameter *J* as a benchmark for having the fastest decay during the dynamics under the non-resonant condition. For larger detuning tending to the dispersive regime 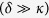, the decay of coherence is instead strongly slowed down^48^. However, as shown in Fig. 5, stationary coherence is forbidden out of resonance when the cavity *C*_2_ is perfect. Since our main aim is the long-time preservation of quantum coherence and thus of entanglement, in the following we only focus on the condition of resonance between qubit and cavity frequencies.

### Two-qubit entanglement preservation

**Figure 6:**
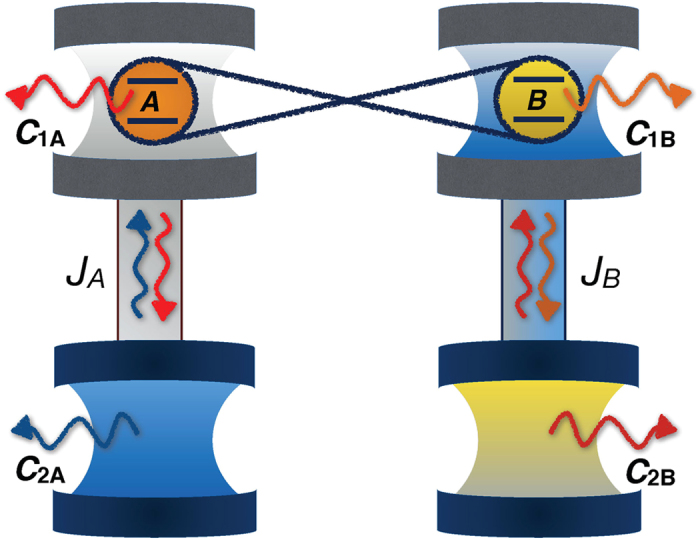
Scheme of the two-qubit architecture. Two independent qubits *A* and *B*, initially entangled, are locally embedded in a cavity *C*_1*j*_ which is in turn coupled to a second cavity *C*_2*j*_ by a coupling strength *J*_*j*_ (*j* = *A*, *B*).

So far, we have studied the manipulation of coherence dynamics of a qubit via an adjustment of coupling strength between two cavities. We now extend this architecture to explore the possibility to harness and preserve the entanglement of two independent qubits, labeled as *A* and *B*. We thus consider *A* (*B*) interacts locally with cavity *C*_1*A*_ (*C*_1*B*_) which is in turn coupled to cavity *C*_2*A*_(*C*_2*B*_) with coupling strength *J*_*A*_ (*J*_*B*_), as illustrated in Fig. 6. That is, we have two independent dynamics with each one consisting of a qubit *j* (*j* = *A*, *B*) and two coupled cavities *C*_1*j*_ − *C*_2*j*_. The total Hamiltonian is then given by the sum of the two independent Hamiltonians, namely, 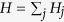, where each *H*_*j*_ is the single-qubit Hamiltonian of Eq. (1). Denoting with Γ_1*j*_ (Γ_2*j*_) the decay rate of cavity *C*_1*j*_ (*C*_2*j*_), we shall assume Γ_1*A*_ = Γ_1*B*_ = Γ as the unit of the other parameters.

As known for the case of independent subsystems, the complete dynamics of the two-qubit system can be obtained by knowing that of each qubit interacting with its own environment^41^,^42^. By means of the single-qubit evolution, we can construct the evolved density matrix of the two atoms, whose elements in the standard computational basis 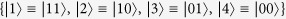 are


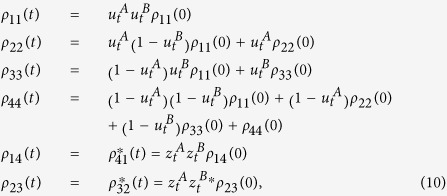


where *ρ*_*lm*_(0) are the density matrix elements of the two-qubit initial state and 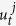, 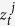 are the time-dependent functions of Eq. (7).

**Figure 7:**
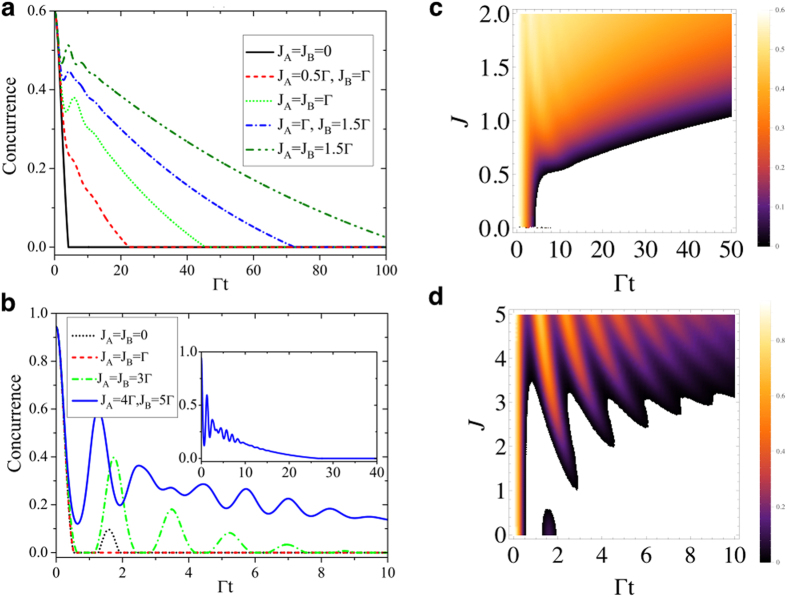
The dynamics of concurrence for different coupling strengths *J*_*A*_ and *J*_*B*_ in (a) weak qubit-cavity coupling regimes with 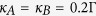 and (b) strong qubit-cavity coupling regimes with 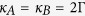. The initial state weights are chosen as (**a**) 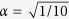, 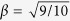 and (**b**) 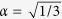, 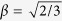, while in both cases 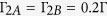. The inset in (b) shows the long-time dynamics of concurrence for 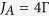 and 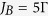. Panels (c,d) show the density plots of the two-qubit concurrence as a function of *J* (*J*_*A*_ = *J*_*B*_ = *J* is here assumed) and scaled time Γ*t*, the others parameters being as in panels (**a**,**b**), respectively. The values of the concurrence in the density plots range within the interval: (**c**) [0, 0.6]; (**d**) [0, 1].

We consider the qubits initially in an entangled state of the form 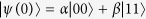 
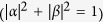
. As is known, this type of entangled states with 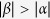 suffers from entanglement sudden death when each atom locally interacts with a dissipative environment^7^,^8^,^9^. As far as non-Markovian environments are concerned, partial revivals of entanglement can occur^38^,^41^,^42^,^43^,^44^,^74^,^75^,^76^,^77^,^78^,^79^,^80^,^81^,^82^,^83^,^84^ typically after asymptotically decaying to zero or after a finite dark period of complete disappearance. It would be useful in practical applications that the non-Markovian oscillations can occur when the entanglement still retain a relatively large value. With our cavity-based architecture, on the one hand we show that the Markovian dynamics of entanglement in the weak coupling regime between the atoms and the corresponding cavities (i.e., *C*_1*A*_ and *C*_1*B*_) can be turned into non-Markovian one by increasing the coupling strengths between the cavities *C*_1*A*_-*C*_2*A*_ and (or) *C*_1*B*_-*C*_2*B*_; on the other hand, we find that the appearance of entanglement revivals can be shifted to earlier times. We employ the concurrence^85^ to quantify the entanglement (see Methods), which for the two-qubit evolved state of Eq. (10) reads CAB   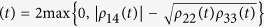. Notice that the concurrence of the Bell-like initial state 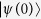 is CAB 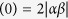. In Fig. 7(a) we plot the dynamics of concurrence CAB  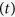 in the weak coupling regime between the two qubits with their corresponding cavities with 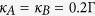 (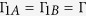 has been assumed). For two-qubit initial states with 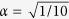, 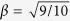, the entanglement experiences sudden death without coupled cavities 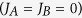. By incorporating the additional cavities with relatively small coupling strength, e.g., *J*_*A*_ = 0.5 Γ and *J*_*B*_ = Γ, the concurrence still undergoes a Markovian decay but the time of entanglement disappearance is prolonged. Increasing the coupling strengths *J*_*A*_, *J*_*B*_ of the relevant cavities drives the entanglement dynamics from Markovian regime to non-Markovian one. Moreover, the entanglement revivals after decay happen shortly after the evolution when the entanglement still has a large value. In general, the concurrences are enhanced pronouncedly with *J*_*A*_ and *J*_*B*_. A comprehensive picture of the dynamics of concurrence as a function of coupling strength *J* is shown in Fig. 7(c) where we have assumed *J*_*A*_ = *J*_*B*_ = *J*. In Fig. 7(b) we plot the dynamics of 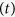 in the strong coupling regime between qubit *j* and its cavity *C*_1*j*_ with 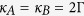 for which the two-qubit dynamics is already non-Markovian in absence of cavity coupling, namely the entanglement can revive after dark periods. Remarkably, the figure shows that when the coupling *J*_*j*_ between *C*_1*j*_ and *C*_2*j*_ is activated and gradually increased in each location, multiple transitions from non-Markovian to Markovian dynamics surface. We point out that the entanglement dynamics within the non-Markovian regime exhibit different qualitative behaviors with respect to the first time when entanglement oscillates. For instance, for 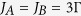, the non-Markovian entanglement oscillations (revivals) happen after its disappearance, while when 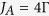 and 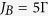 the entanglement oscillates before its sudden death. These dynamical features are clearly displayed in Fig. 7(d).

**Figure 8:**
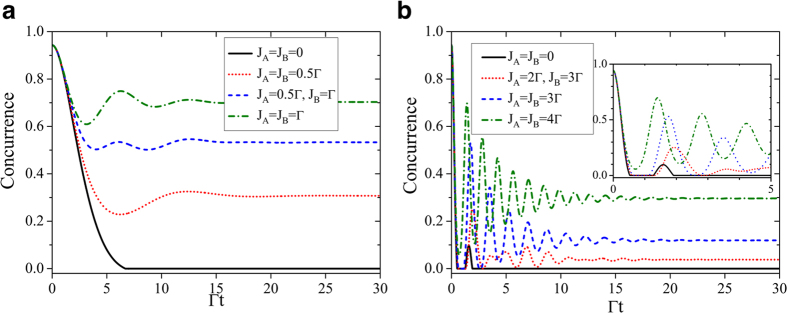
The dynamics of concurrence for different coupling strengths *J-* and *J*_*B*_ in the presence of ideal coupled cavities *C*_2*A*_ and *C*_2*B*_ with 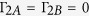 for (a) 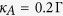, 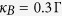 and (b) 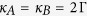. The other parameters are chosen as 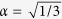, 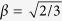. The inset in (**b**) shows the short time dynamics of concurrence.

**Figure 9:**
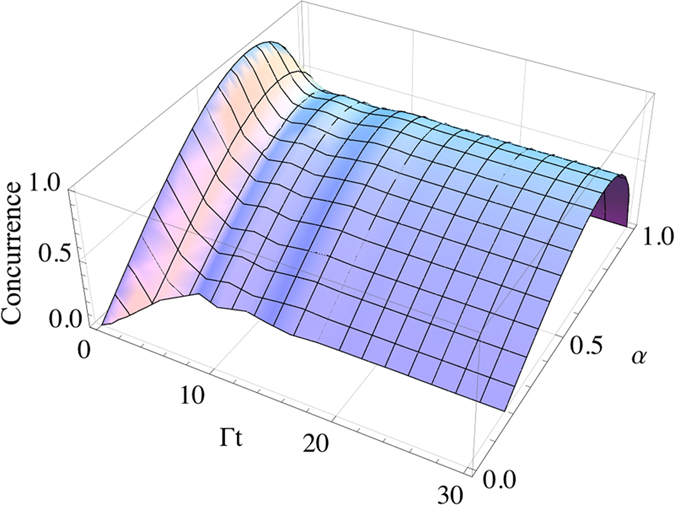
The concurrence as a function of the two-qubit initial state parameter *α* and the scaled time Γ*t* for 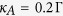, 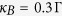,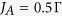,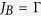 and 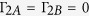. The parameter *α* quantifies the initial entanglement according to the concurrence CAB 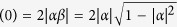.

As expected according to the results obtained before on the single-qubit coherence, a steady concurrence arises in the long-time limit if the secondary cavities *C*_2*A*_, *C*_2*B*_ do not lose photons, i.e., 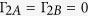. Figure 8(a) shows the dynamics of concurrence for qubits coupled to their cavities with strengths 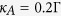, 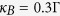. We can readily see that, in absence of coupling with the secondary cavities (*J*_*A*_ = *J*_*B*_ = 0), the entanglement disappear at a finite time without any revival. Contrarily, if the local couplings *C*_1*j*_-*C*_2*j*_ are switched on and increased, the entanglement does not vanish at a finite time any more and reaches a steady value after undergoing non-Markovian oscillations. Furthermore, the steady value of concurrence is proportional to the local cavity coupling strengths *J*_*A*_, *J*_*B*_. In Fig. 8(b), the concurrence dynamics for 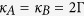 is plotted under which the two-qubit entanglement experiences non-Markovian features, that is revivals after dark periods, already in absence of coupled cavities, as shown by the black solid curve for *J*_*A*_ = *J*_*B*_ = 0. Of course, in this case the entanglement eventually decays to zero. On the contrary, by adjusting suitable nonzero values of the local cavity couplings a considerable amount of entanglement can be trapped. As a peculiar qualitative dynamical feature, we highlight that the entanglement can revive and then be frozen after a finite dark period time of complete disappearance (e.g., see the inset of Fig. 8(b), for the short-time dynamics with 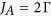, 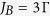 and also 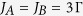). We finally point out that the the amount of preserved entanglement depends on the choice of the initial state (i.e., on the initial amount of entanglement) of the two qubits. As displayed in Fig. 9, the less initial entanglement, the less entanglement is in general maintained in the ideal case of 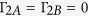. However, since there is not a direct proportionality between the evolved concurrence CAB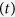 and its initial value CAB 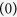, the maximal values of concurrence do not exactly appear at 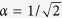 (corresponding to maximal initial entanglement) at any time in the evolution, as instead one could expect. It can be then observed that nonzero entanglement trapping is achieved for *α* > 0.2.

### Experimental paramaters

We conclude our study by discussing the experimental feasibility of the cavity-based architecture here proposed for the two-qubit assembly. Due to its cavity quantum electrodynamics characteristics, our engineered environment finds its natural realization in the well-established framework of circuit quantum electrodynamics (cQED) with transmon qubits and coplanar waveguide cavities^64^,^86^,^87^,^88^,^89^. The entangled qubits can be initialized by using the standard technique of a transmission-line resonator as a quantum bus^64^,^90^. Initial Bell-like states as the one we have considered here can be currently prepared with very high fidelity^90^. Considering up-to-date experimental parameters^86^,^87^,^88^,^89^ applied to our global system of Fig. 6, the average photon decay rate for the cavity *C*_1*j*_ (*j* = *A*, *B*) containing the qubit is 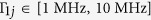, while the average photon lifetime for the high quality factor cavity *C*_2*j*_ is 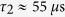
87, which implies 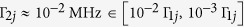. The qubit-cavity interaction intensity *κ*_*j*_ and the cavity-cavity coupling strength *J*_*j*_ are usually of the same order of magnitude, with typical values 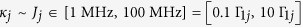. The typical cavity frequency is 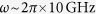
64 while the qubit transition frequency can be arbitrarily adjusted in order to be resonant with the cavity frequency. The above experimental parameters put our system under the condition 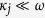 which guarantees the validity of the rotating wave approximation (RWA) for the qubit-cavity interaction here considered in the Hamiltonian of Eq. (1).

In order to assess the extent of entanglement preservation expected under these experimental conditions, we can analyze the concurrence evolution under the same parameters of Fig. 8(a) for *κ*_*j*_, *J*_*j*_, which are already within the experimental values, but with 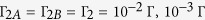 instead of being zero (ideal case), where 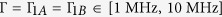. The natural estimated disappearance time of entanglement in absence of coupling between the cavities 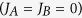 is 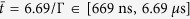, as seen from Fig. 8(a). When considering the experimental achievable decay rates for the cavities *C*_2*j*_, we find that the entanglement is expected to be preserved until times t^*^ orders of magnitude longer than 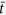, as shown in Table 1. In the case of higher quality factors for the cavities *C*_2*j*_, such that the photon decay rate is of the order of 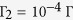, the entanglement can last even until the order of the seconds. These results provide a clear evidence of the practical powerful of our simple two-qubit architecture in significantly extending quantum entanglement lifetime for the implementation of given entanglement-based quantum tasks and algorithms^14^,^90^,^91^,^92^.

It is worth to mention that nowadays cQED technologies are also able to create a qubit-cavity coupling strength comparable to the cavity frequency, thus entering the so-called ultra-strong coupling regime^93^. In that case the RWA is to be relaxed and the counter-rotating terms in the qubit-cavity interaction have to be taken into account. According to known results for the single qubit evolution beyond the RWA^94^, it appears that the main effect of the counter-rotating terms in the Rabi Hamiltonian is the photon creation from vacuum under dephasing noise, which in turns induces a bit-flip error in the qubit evolution. This photon creation would be instead suppressed in the presence of dissipative (damping) mechanisms^94^. Since our cavity-based architecture is subject to amplitude damping noise, the qualitative long-time dynamics of quantum coherence and thus of entanglement are expected not to be significantly modified with respect to the case when RWA is retained. These argumentations stimulate a detailed study of the performance of our proposed architecture under the ultra-strong coupling regime out of RWA, to be addressed elsewhere.

## Discussion

In this work, we have analyzed the possibility to manipulate and maintain quantum coherence and entanglement of quantum systems by means of a simple yet effective cavity-based engineered environment. In particular, we have seen how an environmental architecture made of two coupled lossy cavities enables a switch between Markovian and non-Markovian regimes for the dynamics of a qubit (artificial atom) embedded in one of the cavity. This feature possesses an intrinsic interest in the context of controlling memory effects of open quantum systems. Moreover, if the cavity without qubit has a small photon leakage with respect to the other one, qubit coherence can be efficiently maintained.

We mention that our cavity-based architecture for the single qubit can be viewed as the physical realization of a photonic band gap for the qubit^95^, inhibiting its spontaneous emission. This property, then extended to the case of two independent qubits locally subject to such an engineered environment, has allowed us to show that quantum entanglement can be robustly shielded from decay, reaching a steady-state entanglement in the limit of perfect cavities. The emergence of this steady-state entanglement within our proposed architecture confirms the mechanism of entanglement preservation when the qubit-environment interaction is dissipative: namely, the simultaneous existence of a bound state between the qubit and its local environment and of a non-Markovian dynamics for the qubit^40^. We remark that this condition is here shown to be efficiently approximated within current experimental parameters such as to maintain a substantial fraction of the entanglement initially shared between the qubits during the evolution. Moreover, we highlight that this goal is achieved even if the local reservoir (cavity) embedding the qubit is memoryless, thanks to the exploitation of an additional good-quality cavity suitably coupled to the first one. Specifically, we have found that, by suitably adjusting the control parameter constituted by this local cavity coupling, the entanglement between the separated qubits can be exploited for times orders of magnitude longer than the natural time of its disappearance in absence of the cavity coupling. These times are expected to be long enough to perform various quantum tasks^14^,^90^.

Our long-living quantum entanglement scheme, besides its simplicity, is straightforwardly extendable to many qubits, thus fulfilling the scalability requirement for complex quantum information and computation protocols. The fact that the qubits are independent and noninteracting also allows for the desirable individual operations on each constituent of a quantum hardware. The results of this work provide new insights regarding the control of the fundamental non-Markovian character of open quantum system dynamics and pave the way to further experimental developments towards the realization of devices able to preserve quantum resources.

## Methods

### Functions of the single qubit density matrix

Let us denote with L−1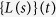 the inverse Laplace transform of *L*(*s*). Then, the functions *u*_*t*_ and *z*_*t*_ appearing in Eq. (7) are expressed as


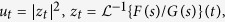


where


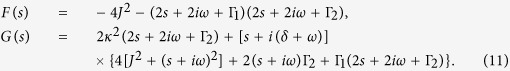


### Entanglement quantification by concurrence

Entanglement for an arbitrary state *ρ*_*AB*_ of two qubits is quantified by concurrence^3^,^85^


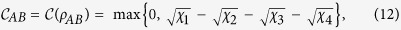


where *χ*_*i*_ 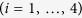 are the eigenvalues in decreasing order of the matrix 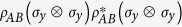, with *σ*_*y*_ denoting the second Pauli matrix and 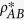 corresponding to the complex conjugate of the two-qubit density matrix *ρ*_*AB*_ in the canonical computational basis 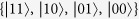.

## Additional Information

**How to cite this article**: Man, Z.-X. *et al.* Cavity-based architecture to preserve quantum coherence and entanglement. *Sci. Rep.* **5**, 13843; doi: 10.1038/srep13843 (2015).

## Figures and Tables

**Table 1:** Estimates of the experimental entanglement lifetimes *t* ^*^ for different values of the second cavities decay rates Γ_2_ and the local cavity couplings *J*_*A*_, *J*_*B*_.

Γ_2_/Γ	*J*_*A*_/Γ = *J*_*B*_/Γ = 0.5	*J*_*A*_/Γ = 0.5, *J*_*B*_/Γ = 1
10^−2^	t^*^ = 454/Γ ∈ [45.4 *μ*s, 454 *μ*s]	t^*^ = 974/Γ ∈ [97.4 *μ*s, 974 *μ*s]
10^−3^	t^*^ = 4481/Γ ∈ [448 *μ*s, 4.48 ms]	t^*^ = 9686/Γ ∈ [0.967 ms, 9.67 ms]

These values are to be compared with the natural entanglement lifetime without cavity coupling, 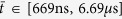. The reference unit 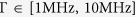.

## References

[b1] Nielsen M. A. & Chuang I. L. Quantum Computation and Quantum Information (Cambridge University Press, Cambridge, 2000).

[b2] Benenti G., Casati G. & Strini G. Principles of quantum computation and information (World Scientific, Singapore, 2007).

[b3] Amico L., Fazio R., Osterloh A. & Vedral V. Entanglement in many-body systems. Rev. Mod. Phys. 80, 517–576 (2008).

[b4] Yu T. & Eberly J. H. Finite-time disentanglement via spontaneous emission. Phys. Rev. Lett. 93, 140404 (2004). 15524773 10.1103/PhysRevLett.93.140404

[b5] Yu T. & Eberly J. H. Quantum open system theory: Bipartite aspects. Phys. Rev. Lett. 97, 140403 (2006). 17155224 10.1103/PhysRevLett.97.140403

[b6] Dodd P. J. & Halliwell J. J. Disentanglement and decoherence by open system dynamics. Phys. Rev. A 69, 052105 (2004).

[b7] Santos M. F., Milman P., Davidovich L. & Zagury M. Direct measurement of finite-time disentanglement induced by a reservoir. Phys. Rev. A 73, 040305 (2006).

[b8] Yu T. & Eberly J. H. Sudden death of entanglement. Science 323, 598–601 (2009). 19179521 10.1126/science.1167343

[b9] Almeida M. P. *et al.* Environment-induced sudden death of entanglement. Science 316, 579 (2007). 17463284 10.1126/science.1139892

[b10] Laurat J., Choi K. S., Deng H., Chou C. W. & Kimble H. J. Heralded entanglement between atomic ensembles: preparation, decoherence, and scaling. Phys. Rev. Lett. 99, 180504 (2007). 17995390 10.1103/PhysRevLett.99.180504

[b11] Eberly J. H. & Yu T. The end of an entanglement. Science 316, 555 (2007). 17463278 10.1126/science.1142654

[b12] Salles A. *et al.* Experimental investigation of the dynamics of entanglement: Sudden death, complementarity, and continuous monitoring of the environment. Phys. Rev. A 78, 022322 (2008).

[b13] Aolita L., de Melo F. & Davidovich L. Open-system dynamics of entanglement: a key issues review. Rep. Prog. Phys. 78, 042001 (2015). 25811809 10.1088/0034-4885/78/4/042001

[b14] Ladd T. D. *et al.* Quantum computers. Nature 464, 45 (2010). 20203602 10.1038/nature08812

[b15] Xiang Z.-L., Ashhab S., You J. & Nori F. Hybrid quantum circuits: Superconducting circuits interacting with other quantum systems. Rev. Mod. Phys. 85, 623 (2013).

[b16] Bennett C. H. *et al.* Purification of noisy entanglement and faithful teleportation via noisy channels. Phys. Rev. Lett. 76, 722 (1996). 10061534 10.1103/PhysRevLett.76.722

[b17] Bennett C. H., Bernstein H. J., Popescu S. & Schumacher B. Concentrating partial entanglement by local operations. Phys. Rev. A 53, 2046 (1996). 9913106 10.1103/physreva.53.2046

[b18] Pan J. W., Gasparoni S., Ursin R., Weihs G. & Zeilinger A. Experimental entanglement purification of arbitrary unknown states. Nature 423, 417 (2003). 12761543 10.1038/nature01623

[b19] Kwiat P. G., Barraza-Lopez S., Stefanov A. & Gisin N. Experimental entanglement distillation and hidden non-locality. Nature 409, 1014 (2001). 11234004 10.1038/35059017

[b20] Dong R. *et al.* Experimental entanglement distillation of mesoscopic quantum states. Nature Phys. 4, 919 (2008).

[b21] Zanardi P. & Rasetti M. Noiseless quantum codes. Phys. Rev. Lett. 79, 3306 (1997).

[b22] Lidar D. A., Chuang I. & Whaley K. B. Decoherence-free subspaces for quantum computation. Phys. Rev. Lett. 81, 2594 (1998). 10.1103/PhysRevLett.85.1758 10970607

[b23] Kwiat P. G., Berglund A. J., Alterpeter J. B. & White A. G. Experimental entanglement distillation and hidden non-locality. Science 290, 498 (2000).11039926

[b24] Maniscalco S., Francica F., Zaffino R. L., Gullo N. L. & Plastina F. Protecting entanglement via the quantum Zeno effect. Phys. Rev. Lett. 100, 090503 (2008). 18352687 10.1103/PhysRevLett.100.090503

[b25] An N. B., Kim J. & Kim K. Nonperturbative analysis of entanglement dynamics and control for three qubits in a common lossy cavity. Phys. Rev. A 82, 032316 (2010).

[b26] Facchi P., Lidar D. A. & Pascazio S. Unification of dynamical decoupling and the quantum Zeno effect. Phys. Rev. A 69, 032314 (2004).

[b27] Shor P. W. Scheme for reducing decoherence in quantum computer memory. Phys. Rev. A 52, 2493(R) (1995). 10.1103/physreva.52.r2493 9912632

[b28] Steane A. M. Error correcting codes in quantum theory. Phys. Rev. Lett. 77, 793 (1996). 10062908 10.1103/PhysRevLett.77.793

[b29] Steane A. M. Multiple-particle interference and quantum error correction. Proc. R. Soc. London A 452, 2551 (1996).

[b30] Calderbank A. R. & Shor P. W. Good quantum error-correcting codes exist. Phys. Rev. A 54, 1098 (1996). 9913578 10.1103/physreva.54.1098

[b31] Sainz I. & Bjork G. Good quantum error-correcting codes exist. Phys. Rev. A 77, 052307 (2008).

[b32] Mukhtar M., Saw T. B., Soh W. T. & Gong J. Universal dynamical decoupling: Two-qubit states and beyond. Phys. Rev. A 81, 012331 (2010).

[b33] Mukhtar M., Soh W. T., Saw T. B. & Gong J. Protecting unknown two-qubit entangled states by nesting Uhrig’s dynamical decoupling sequences. Phys. Rev. A 82, 052338 (2010).

[b34] Wang Z.-Y. & Liu R.-B. Protection of quantum systems by nested dynamical decoupling. Phys. Rev. A 83, 022306 (2011).

[b35] Pan Y., R-Xi Z. & Gong J. Optimized dynamical decoupling sequences in protecting two-qubit states. J. Phys. B: At. Mol. Opt. Phys. 44, 175501 (2011).

[b36] Lo Franco R., D’Arrigo A., Falci G., Compagno G. & Paladino E. Preserving entanglement and nonlocality in solid-state qubits by dynamical decoupling. Phys. Rev. B 90, 054304 (2014).

[b37] Lo Franco R., D’Arrigo A., Falci G., Compagno G. & Paladino E. Spin-echo entanglement protection from random telegraph noise. Phys. Scr. T153, 014043 (2013).

[b38] Lo Franco R., Bellomo B., Maniscalco S. & Compagno G. Dynamics of quantum correlations in two-qubit systems within non-Markovian environments. Int. J. Mod. Phys. B 27, 1345053 (2013).

[b39] Tan J., Kyaw T. H. & Yeo Y. Non-Markovian environments and entanglement preservation. Phys. Rev. A 81, 062119 (2010).

[b40] Tong Q. J., An J. H., Luo H. G. & Oh C. H. Mechanism of entanglement preservation. Phys. Rev. A 81, 052330 (2010).

[b41] Bellomo B., Lo Franco R. & Compagno G. Non-Markovian effects on the dynamics of entanglement. Phys. Rev. Lett. 99, 160502 (2007). 17995229 10.1103/PhysRevLett.99.160502

[b42] Bellomo B., Lo Franco R. & Compagno G. Entanglement dynamics of two independent qubits in environments with and without memory. Phys. Rev. A 77, 032342 (2008).

[b43] Lo Franco R., Bellomo B., Andersson E. & Compagno G. Revival of quantum correlation without system-environment back-action. Phys. Rev. A 85, 032318 (2012).

[b44] Xu J.-S. *et al.* Experimental recovery of quantum correlations in absence of system-environment back-action. Nature Commun. 4, 2851 (2013). 24287554 10.1038/ncomms3851 PMC3868330

[b45] D’Arrigo A., Lo Franco R., Benenti G., Paladino E. & Falci G. Recovering entanglement by local operations. Ann. Phys. 350, 211 (2014). 10.1038/srep08575 PMC4339803 25712406

[b46] Orieux A. *et al.* Experimental on-demand recovery of quantum entanglement by local operations within non-Markovian dynamics. Sci. Rep. 5, 8575 (2015). 25712406 10.1038/srep08575 PMC4339803

[b47] Bellomo B., Lo Franco R., Maniscalco S. & Compagno G. Entanglement trapping in structured environments. Phys. Rev. A 78, 060302(R) (2008).

[b48] Bellomo B., Lo Franco R., Maniscalco S. & Compagno G. Two-qubit entanglement dynamics for two different non-Markovian environments. Phys. Scr. T140, 014014 (2010).

[b49] Lodahl P. *et al.* Controlling the dynamics of spontaneous emission from quantum dots by photonic crystals. Nature 430, 654 (2004). 15295594 10.1038/nature02772

[b50] Zhu S. Y. & Scully M. O. Spectral line elimination and spontaneous emission cancellation via quantum interference. Phys. Rev. Lett. 76, 388 (1996). 10061444 10.1103/PhysRevLett.76.388

[b51] Scully M. O. & Zhu S. Y. Quantum control of the inevitable. Science 281, 1973 (1998).

[b52] Das S. & Agarwal G. S. Protecting bipartite entanglement by quantum interferences. Phys. Rev. A 81, 052341 (2010).

[b53] Kim Y. S., Lee J. C., Kwon O. & Kim Y. H. Protecting entanglement from decoherence using weak measurement and quantum measurement reversal. Nature Phys. 8, 117 (2012).

[b54] Man Z. X., Xia Y. J. & An N. B. Manipulating entanglement of two qubits in a common environment by means of weak measurements and quantum measurement reversals. Phys. Rev. A 86, 012325 (2012).

[b55] Man Z. X., Xia Y. J. & An N. B. Enhancing entanglement of two qubits undergoing independent decoherences by local pre- and postmeasurements. Phys. Rev. A 86, 052322 (2012).

[b56] Benatti F., Floreanini R. & Piani M. Environment induced entanglement in Markovian dissipative dynamics. Phys. Rev. Lett. 91, 070402 (2003). 12934997 10.1103/PhysRevLett.91.070402

[b57] Scala M., Migliore R., Messina A. & Sánchez-Soto L. L. Robust stationary entanglement of two coupled qubits in independent environments. Eur. Phys. J. D 61, 199 (2011).

[b58] Brask J. B., Brunner N., Haack G. & Huber M. Autonomous quantum thermal machine for generating steady-state entanglement. *Preprint at arXiv:1504.00187* (2015).

[b59] Plenio M. B. & Huelga S. F. Entangled light from white noise. Phys. Rev. Lett. 88, 197901 (2002). 12005665 10.1103/PhysRevLett.88.197901

[b60] Hartmann L., Dü W. & Briegel H. J. Entanglement and its dynamics in open dissipative systems. New J. Phys. 9, 230 (2007).

[b61] Bellomo B. & Antezza M. Creation and protection of entanglement in systems out of thermal equilibrium. New J. Phys. 15, 113052 (2013).

[b62] Bellomo B. & Antezza M. Steady entanglement out of thermal equilibrium. EPL (Europhysics Letters) 104, 10006 (2013).

[b63] Huelga S. F., Rivas Á. & Plenio M. B. Non-Markovianity-assisted steady state entanglement. Phys. Rev. Lett. 108, 160402 (2012). 22680702 10.1103/PhysRevLett.108.160402

[b64] Blais A., Huang R.-S., Wallraff A., Girvin S. M. & Schoelkopf R. J. Cavity quantum electrodynamics for superconducting electrical circuits: an architecture for quantum computation. Phys. Rev. A 69, 062320 (2004).

[b65] Breuer H.-P. & Petruccione F. The Theory of Open Quantum Systems (Oxford University Press, Oxford, New York, 2002).

[b66] Garraway B. M. Nonperturbative decay of an atomic system in a cavity. Phys. Rev. A 55, 2290 (1997).

[b67] Garraway B. M. Decay of an atom coupled strongly to a reservoir. Phys. Rev. A 55, 4636 (1997).

[b68] Baumgratz T., Cramer M. & Plenio M. B. Quantifying coherence. Phys. Rev. Lett. 113, 140401 (2014). 25325620 10.1103/PhysRevLett.113.140401

[b69] Breuer H.-P., Laine E.-M. & Piilo J. Measure for the degree of non-Markovian behavior of quantum processes in open systems. Phys. Rev. Lett. 103, 210401 (2009). 20366019 10.1103/PhysRevLett.103.210401

[b70] Lorenzo S., Plastina F. & Paternostro M. Geometrical characterization of non-Markovianity. Phys. Rev. A 88, 020102(R) (2013).

[b71] Rivas Á., Huelga S. F. & Plenio M. B. Entanglement and non-Markovianity of quantum evolutions. Phys. Rev. Lett. 105, 050403 (2010). 20867898 10.1103/PhysRevLett.105.050403

[b72] Bylicka B., Chruściński D. & Maniscalco S. Non-Markovianity and reservoir memory of quantum channels: a quantum information theory perspective. Sci. Rep. 4, 5720 (2014). 25043763 10.1038/srep05720 PMC4104480

[b73] Man Z.-X., Xia Y.-J. & Lo Franco R. Harnessing non-Markovian quantum memory by environmental coupling. Phys. Rev. A 92, 012315 (2015).

[b74] Lo Franco R., D’Arrigo A., Falci G., Compagno G. & Paladino E. Entanglement dynamics in superconducting qubits affected by local bistable impurities. Phys. Scr. T147, 014019 (2012).

[b75] D’Arrigo A., Lo Franco R., Benenti G., Paladino E. & Falci G. Hidden entanglement, system-environment information flow and non-Markovianity. Int. J. Quantum Inf. 12, 1461005 (2014).

[b76] D’Arrigo A., Lo Franco R., Benenti G., Paladino E. & Falci G. Hidden entanglement in the presence of random telegraph dephasing noise. Phys. Scr. T153, 014014 (2013).

[b77] Bellomo B., Lo Franco R. & Compagno G. Dynamics of non-classically-reproducible entanglement. Phys. Rev. A 78, 062309 (2008).

[b78] Ban M., Kitajima S. & Shibatay F. Decoherence of quantum information in the non-Markovian qubit channel. J. Phys. A: Math. Gen. 38, 7161 (2005).

[b79] Ban M. Decoherence of continuous variable quantum information in non-Markovian channels. J. Phys. A: Math. Gen. 39, 1927 (2006).

[b80] Liu K.-L. & Goan H.-S. Non-Markovian entanglement dynamics of quantum continuous variable systems in thermal environments. Phys. Rev. A 76, 022312 (2007).

[b81] Yonac M., Yu T. & Eberly J. H. Sudden death of entanglement of two Jaynes-Cummings atoms. J. Phys. B: At. Mol. Opt. Phys. 39, S621 (2006).

[b82] Man Z. X., Xia Y. J. & An N. B. Entanglement measure and dynamics of multiqubit systems: non-Markovian versus Markovian and generalized monogamy relations. New J. Phys. 12, 033020 (2010).

[b83] Bai Y. K., Xu Y. F. & Wang Z. D. General monogamy relation for the entanglement of formation in multiqubit systems. Phys. Rev. Lett. 113, 100503 (2014). 25238341 10.1103/PhysRevLett.113.100503

[b84] Bai Y. K., Ye M. Y. & Wang Z. D. Entanglement monogamy and entanglement evolution in multipartite systems. Phys. Rev. A 80, 044301 (2009).

[b85] Wootters W. K. Entanglement of formation of an arbitrary state of two qubits. Phys. Rev. Lett. 80, 2245–2248 (1998).

[b86] Bronn N. T. *et al.* Reducing spontaneous emission in circuit quantum electrodynamics by a combined readout/filter technique. *Preprint at arXiv:1504.04353* (2015).

[b87] Vlastakis B. *et al.* Violating Bell’s inequality with an artificial atom and a cat state in a cavity. *Preprint at arXiv:1504.02512* (2015).

[b88] Leek P. J. *et al.* Cavity quantum electrodynamics with separate photon storage and qubit readout modes. Phys. Rev. Lett. 104, 100504 (2010). 20366408 10.1103/PhysRevLett.104.100504

[b89] FinkJ. M. *et al.* Climbing the Jaynes-Cummings ladder and observing its 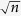 nonlinearity in a cavity QED system. Nature 454, 315 (2008).1863341310.1038/nature07112

[b90] DiCarlo L. *et al.* Demonstration of two-qubit algorithms with a superconducting quantum processor. Nature 460, 240 (2009). 19561592 10.1038/nature08121

[b91] Horodecki R., Horodecki P., Horodecki M. & Horodecki K. Quantum entanglement. Rev. Mod. Phys. 81, 865–942 (2009).

[b92] Brunner N., Cavalcanti D., Pironio S., Scarani V. & Wehner S. Bell nonlocality. Rev. Mod. Phys. 86, 419 (2014).

[b93] Niemczyk T. *et al.* Circuit quantum electrodynamics in the ultrastrong-coupling regime. Nature Phys. 6, 772–776 (2010).

[b94] Werlang T., Dodonov A. V., Duzzioni E. I. & Villas-Bôas C. J. Rabi model beyond the rotating-wave approximation: Generation of photons from vacuum through decoherence. Phys. Rev. A 78, 053805 (2008).

[b95] Mazzola L., Maniscalco S., Piilo J., Suominen K.-A. & Garraway B. M. Pseudomodes as an effective description of memory: Non-Markovian dynamics of two-state systems in structured reservoirs. Phys. Rev. A 80, 012104 (2009).

